# Dexamethasone does not ameliorate gliosis in a mouse model of neurodegenerative disease

**DOI:** 10.1016/j.bbrep.2020.100817

**Published:** 2020-09-24

**Authors:** Xiaolian Ye, Gang Zou, Jinxing Hou, Huiru Bi, Cuihua Zhou, Runmin Wang, Yun Xu, Chun Wang, Guiquan Chen, Zhenyu Yin, Jinping Zhang, Chaoli Huang

**Affiliations:** a School of Medicine, MOE Key Laboratory of Model Animal for Disease Study, Model Animal Research Center, Nanjing University, 12 Xuefu Avenue, Nanjing, Jiangsu Province, 210061, China; b Department of Anesthesiology, The Second Affiliated Changzhou People's Hospital of Nanjing Medical University, Changzhou, Jiangsu, 213000, China; c Department of Neurology, Jiangsu Provincial Key Medical Discipline, Nanjing Drum Tower Hospital, Nanjing University Medical School, 321 Zhongshan Avenue, Nanjing, Jiangsu Province, 210008, China; d Department of Geriatric, Nanjing Drum Tower Hospital, Nanjing University Medical School, 321 Zhongshan Avenue, Nanjing, Jiangsu Province, 210008, China; e Department of Medicament, Nanjing Drum Tower Hospital, Nanjing University Medical School, 321 Zhongshan Avenue, Nanjing, Jiangsu Province, 210008, China

**Keywords:** Neurodegeneration, Neuroinflammation, Dexamethasone, Neuron, Synaptic loss

## Abstract

Prolonged neuroinflammation is a driving force for neurodegenerative disease, and agents against inflammatory responses are regarded as potential treatment strategies. Here we aimed to evaluate the prevention effects on gliosis by dexamethasone (DEX), an anti-inflammation drug. We used DEX to treat the *nicastrin* conditional knockout (cKO) mouse, a neurodegenerative mouse model. DEX (10 mg/kg) was given to 2.5-month-old *nicastrin* cKO mice, which have not started to display neurodegeneration and gliosis, for 2 months. Immunohistochemistry (IHC) and Western blotting techniques were used to detect changes in neuroinflammatory responses. We found that activation of glial fibrillary acidic protein (GFAP) positive or ionized calcium binding adapter molecule1 (Iba1) positive cells was not inhibited in *nicastrin* cKO mice treated with DEX as compared to those treated with saline. These data suggest that DEX does not prevent or ameliorate gliosis in a neurodegenerative mouse model when given prior to neuronal or synaptic loss.

## Highlights

• Dexamethasone does not affect astrocyte activation in *NCT* cKO mice.
• Dexamethasone does not affect apoptosis in *NCT* cKO mice.
• Dexamethasone does not affect dendrites or synapses in *NCT* cKO mice.

## 1 Introduction

Neurodegenerative disease (ND) is mainly classified as Alzheimer's disease (AD), Parkinson's disease, Huntington's disease, frontotemporal dementia and amyotrophic lateral sclerosis [1]. ND is characterized by progressive neuronal loss and abnormal protein assemblies [[2], [3], [4]]. Whereas the etiology of ND is still not clear, it is well believed that neuroinflammation plays a pivotal role [[5], [6], [7]]. Two types of glial cells including astrocytes and microglia are involved in neuroinflammation, accompanied with changes in cytokines and chemokines such as interleukin 1β (IL1β) [8], IL6 [9], tumor necrosis factor α (TNFα) [10] and transforming growth factor β (TGFβ) [11]. Normal physiological functions of astrocytes are to stabilize neurons, form blood-brain barrier and regulate synaptic plasticity [12]. Astrocytes can be activated from the resting state if the brain gets lesioned under normal and diseased conditions. Reactive astrocytes are characterized by increased expression of glial fibrillary acidic protein (GFAP), and are widely observed in animal models of NDs [13]. Astrocytes can secrete a variety of cytokines which promote inflammatory responses [5].

Microglia can also be activated by neuronal loss and protein aggregates in the central nervous system (CNS), and can migrate to the site of injury to initiate a series of immune responses [5]. Activated microglia release a series of degradation enzymes such as insulin degrading enzyme and neprilysin to degrade fibrous Aβ in the pathology of AD [14]. Overall, neuroinflammation associated with astrogliosis or microgliosis is an important pathological feature of ND.

Abundant evidence has shown that anti-inflammatory drugs may exhibit beneficial effects on neuroinflammation in ND [[15], [16], [17]]. DEX is a steroid that inhibits the expression of several immune mediators [18]. Indeed, dexamethasone (DEX) treatment could reduce the production of pro-inflammatory cytokines including IL1 and TNFα [19], and inhibit microglial ramification and proliferation in vitro [20]. A previous study reported that DEX plays a neuroprotective role through inhibition on microgliosis via expression of microglial lipocortin [21]. Overall, the above evidence suggests that DEX may be a potential agent to inhibit inflammatory responses and to protect neurons. However, effects of DEX on neuroinflammation in ND models remain largely uninvestigated.

The γ–secretase complex is composed of four subunits including presenilin (PS), nicastrin, presenilin enhancer 2 and anterior pharynx defective 1 (Aph-1) [22]. Accumulating evidence has demonstrated that forebrain neuron specific deletion of γ–secretase subunits leads to age-dependent neurodegeneration [[23], [24], [25], [26], [27], [28]]. Consistent with these findings, our previous work has shown that loss of nicastrin function causes age-dependent cortical neuron loss and striking neuronal inflammatory responses in mice [29]. In this study, we examined effects of DEX on neuroinflammation in *nicastrin* cKO mice. However, no significant effects on the number of GFAP+ and ionized calcium binding adapter molecule1 (Iba1)+ cells were observed in DEX-treated *nicastrin* cKO mice as compared to those treated by saline. These findings suggest that long-term treatment of DEX may not be effective to ameliorate gliosis in ND.

## 2 Materials and methods

### 2.1 Animals

Floxed *nicastrin* (*nicastrin* ^*f/f*^) and *calcium/calmodulin-dependent protein kinase α-Cre* (*CaMKIIα*-*Cre*) transgenic (Tg) mice were described previously [15,[28], [29], [30], [31]]. To generate forebrain specific *nicastrin* cKO mice, *nicastrin* ^*f/f*^ were first crossed with *CaMKIIα*-*Cre* to obtain *nicastrin* ^*f/+*^;*CaMKIIα*-*Cre*. The latter were bred to *nicastrin* ^*f/f*^ to get age-matched *nicastrin* ^*f/f*^ (control) and *nicastrin* ^*f/f*^;*CaMKIIα-Cre* (*nicastrin* cKO) for experiments.

The genetic background of the mice used here was C57BL/6. Mice were housed in an SPF room of the core animal facility of the Model Animal Research Center (MARC) at Nanjing University. The room temperature was kept at 25 ± 1 °C. The light-cycle was automatically controlled (12 h for light and 12 h for dark). Animals had free access to food and water. Mouse breeding was conducted under an IACUC-approved animal protocol in Nanjing University. The experimental protocol was approved by the institutional committee of the MARC at Nanjing University.

### 2.2 DEX treatment

DEX was purchased from Sangon Biotech (BBI A601187) [18]. The concentration of DEX for this study was 10 mg/kg for each mouse [32]. DEX was freshly prepared before injection. Mice received intraperitoneal injection of DEX in saline (the DEX group) or saline alone (the saline group) every two days for 2 months. Mice were sacrificed 24 h after the final injection and brains were dissected.

### 2.3 Immunohistochemistry (IHC)

Mice were perfused with phosphate buffer solution (PBS). The brain was dissected out and then fixed in 4% paraformaldehyde (PFA) overnight. After the fixation, the brain was washed using PBS for several times. Brains were dehydrated and then embedded in paraffin. Paraffin blocks were sectioned at the thickness of 10 μm. For IHC experiments, sagittal sections were deparaffinized, ethanol hydrated. After antigen retrieval with 0.01 M sodium citrate and blocking catalase with 30% hydrogen peroxide, sections were incubated with BSA (5% bovine serum albumin in PBS for 30 min), and then incubated with monoclonal antibodies overnight. The slides were rinsed with PBS for several times to wash out the first primary antibody. After incubation with secondary antibodies diluted in PBS, the sections were incubated with the ABC (avidin-peroxidase complex) kit (Vector). After the reaction with DAB (Diaminobenzidine) (Vector), sections were dehydrated by ethanol and xylene, and then mounted using neutral resin. Primary antibodies used were as following: *anti*-GFAP (1:500; Sigma-Aldrich), *anti*-NeuN (neuronal nuclei) (1:500; Millipore), *anti*-SVP38 (synaptophysin) (1:500; Sigma-Aldrich), and anti-MAP2 (microtubule assoicated protein 2) (1:500; Sigma-Aldrich). For fluorescence IHC, the following secondary antibodies were used: Alexa Fluor 488 goat anti-mouse and Alexa Fluor 594 goat anti-mouse (Invitrogen). The dilution of the second antibody was 1:500. Sections were scanned and analyzed using an Olympus BX53-CellSens Standard system.

### 2.4 Tissue preparation

Mouse cortices were dissected and homogenized in cold radio immunoprecipitation assay lysis buffer containing protease and phosphatase inhibitors. Lysates were cleared by centrifugation (12,000 rpm for 25 min).

### 2.5 Immunoblotting

Western blotting was conducted using a protocol described previously [3,33]. Normalized volumes of samples (40 μg total protein) were resolved in 10% 15-well SDS-PAGE gels (invitrogen), transferred to nitrocellulose membrane. After blocking with 5% (w/v) dry milk for 1 h, membranes were probed with primary antibodies overnight. The membrane was washed using TBS for three times, and then incubated with infrared dye-coupled secondary antibodies. Membranes were scanned using Odyssey Infrared Imaging System (Li-Cor). Primary antibodies used were as following: *anti*-nicastrin (1:500; Sigma-Aldrich), *anti*-β-actin (1:10,000; SAB, College Park, MA, USA) and anti-APP (1:1000; Sigma-Aldrich). Secondary antibodies such as goat anti-rabbit IRdye800, goat anti-rabbit IRdye680, goat anti-mouse IRdye800 and goat anti-mouse IRdye680 were included.

### 2.6 Cell counting

Three sagittal sections spaced 40 μm apart were used for IHC for each mouse. IHC images for GFAP, Iba1 or NeuN were captured using the Olympus BX53- CellSens Standard system. Images were taken under the 40× objective lens of the Olympus B×53 microscope. For counting on GFAP+ and Iba1+ cells, we captured images randomly from five different areas in each brain section. GFAP+ and Iba1+ cells were counted for each microscopy field and averaged across fields using methods described previously [29,31]. For NeuN + cell counting, images were randomly captured for three distinct cortical areas in each section, each cortical area being 40 × 40 μm^2^. NeuN + cells were then averaged for each area across sections.

### 2.7 Statistical analysis

Data were presented as the mean ± SEM. ANOVA was performed to examine the difference between control and cKO mice. *P* < 0.05 (*) was considered statistically significant. For all cell counting experiments, at least three mice per group were used [23].

## 3 Results

### 3.1 The effect of DEX on body weight

**Fig. 1:**
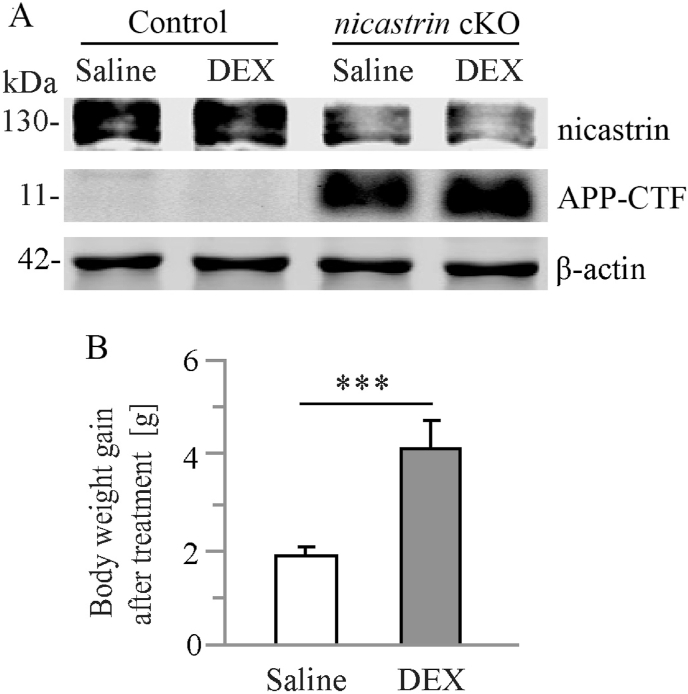
**Molecular analysis on neuron specific *nicastrin* cKO mice**. **A.** Western blotting on nicastrin and APP-CTF. Cortical samples for 4.5-month control and *nicastrin* cKO mice were used. **B.** Body weight of saline-treated and DEX-treated mice after 2-month treatment period (***, p < 0.005).

Previous evidence has shown that forebrain specific *PS1/2*, *nicastrin* or *Aph-1* conditional knockout mice exhibit age-related neuron loss [23,25,26,28,29,34], making them excellent ND models for drug efficacy testing. In this study, we aimed to use neuron-specific *nicastrin* cKO mice to test the effect of DEX on age-dependent neuronal loss. In this model, Cre recombinase is expressed specifically in excitatory neurons of the forebrain since the age of 1.5–2 months [15,24,29]. The animals were treated by DEX for 2 months since the age of 2.5 months. Our molecular analysis revealed that levels of nicastrin were significantly reduced and levels of the c-terminal fragment of amyloid precursor protein (APP-CTF) were increased in the cortex of the cKO mice (Fig. 1A).

After two-month treatment with DEX, we measured changes on body weight of the mice tested. We observed significant increase on the body weight gain in DEX-treated mice as compared to those receiving saline (Fig. 1B: p < 0.005; n = 6 per group), suggesting that long treatment of DEX may increase the body weight. This finding was consistent with previous reports [35,36].

### 3.2 The effect of DEX on gliosis in nicastrin cKO mice

**Fig. 2:**
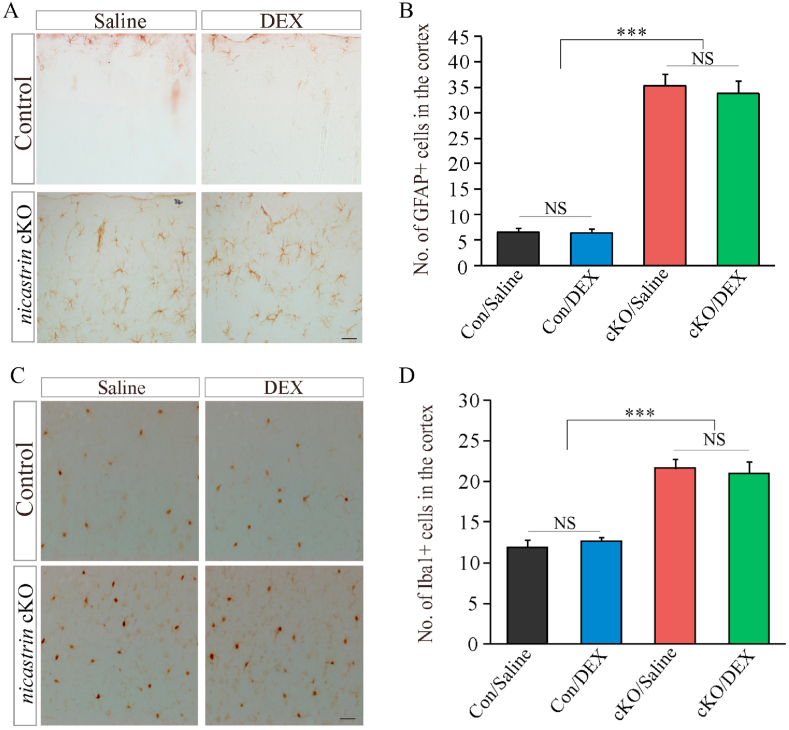
**The effect of DEX on gliosis in *nicastin* cKO mice**. **A.** IHC for GFAP in the cortices of saline- or DEX-treated control and *nicastrin* cKO mice at 4.5 month. The 200 μm × 200 μm images were taken for cell counting. Scale bar = 20 μm. **B.** Cell-counting results for GFAP + cells in cortices of saline- or DEX-treated control and *nicastrin* cKO mice (NS, not significant; ***, p < 0.005). **C.** IHC for Iba1 in the cortices of saline- or DEX-treated control and *nicastrin* cKO mice at 4.5 month. The 200 μm × 200 μm images were taken for cell counting. Scale bar = 20 μm. **D.** Cell-counting result for Iba1+ cells in cortices of saline- or DEX-treated control and *nicastrin* cKO mice (NS, not significant; ***, p < 0.005).

Following a 2-month period of DEX treatment, we analyzed astrogliosis by performing IHC on GFAP. We observed significantly increased number of GFAP + cells in *nicastrin* cKO cortices at 4.5 months as compared to controls (Fig. 2A and B: p < 0.005; n = 3–4 per group). However, we did not observe significant difference on the number of GFAP + cells between saline-treated and DEX-treated control cortices or between saline-treated and DEX-treated *nicastrin* cKO cortices (Fig. 2A and B: p > 0.1; n = 3–4 per group). These results suggest that DEX does not reduce astrogliosis in *nicastrin* cKO mice.

To examine whether DEX affected microgliosis, we performed IHC on Iba1. We found increased number of Iba1+ cells in *nicastrin* cKO cortices at 4.5 months as compared to controls (Fig. 2C and D: p < 0.005; n = 3 per group), but no significant difference on the number of Iba1+ cells saline-treated and DEX-treated control cortices or between saline-treated and DEX-treated *nicastrin* cKO cortices (Fig. 2C and D: p > 0.1; n = 3 per group). These results suggest that 2-month treatment of *nicastrin* cKO mice with DEX does not inhibit microgliosis.

### 3.3 The effect of DEX on the number of neurons and apoptosis in nicastrin cKO mice

**Fig. 3:**
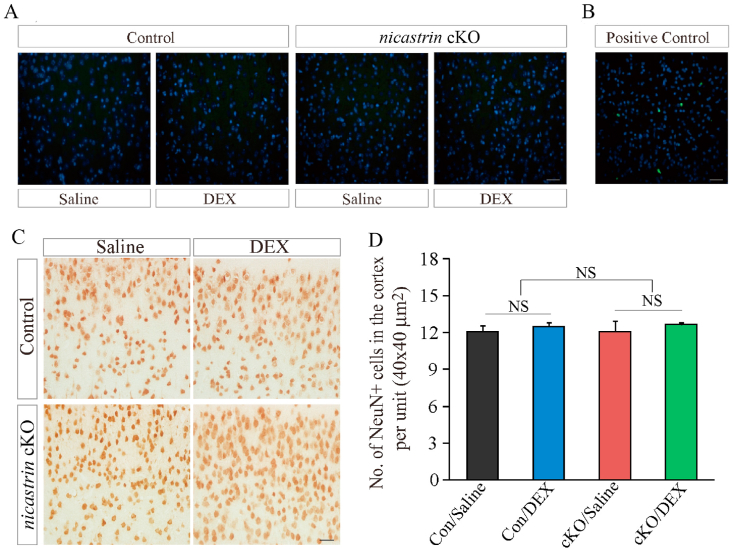
**The effect of DEX on apoptosis and neuronal loss in *nicastrin* cKO mice**. **A.** TUNEL staining on brain sections of saline- or DEX-treated control and *nicastrin* cKO mice at 4.5 months. Scale bar = 20 μm. **B.** Brain section from a 4-month-old *Dicer* cKO mouse was used as the positive control. Scale bar = 20 μm. **C.** IHC for NeuN in the cortices of saline- or DEX-treated control and *nicastrin* cKO mice at 4.5 month. A 40 μm × 40 μm area were taken for cell counting. Scale bar = 20 μm. **D.** Cell-counting results for NeuN + cells in cortices of saline- or DEX-treated control and *nicastrin* cKO mice (NS, not significant).

Previous evidence showed that DEX induces apoptosis in the brain [37]. To examine the effect of DEX on apoptosis in *nicastrin* cKO mice, we performed the terminal deoxynucleotidyl transferase-mediated dUTP-biotin nick end labeling (TUNEL) experiment. However, we found that TUNEL + cells were not detected in DEX-treated *nicastrin* cKO and control mice (Fig. 3A and B).

To examine the number of cortical neurons in 4.5 months old *nicastrin* cKO mice, we performed IHC on NeuN. We observed no significant difference on the total number of NeuN + cells between control and *nicastrin* cKO mice at 4.5 month (Fig. 3C and D: p > 0.1; n = 3 per group). Overall, these results suggest no neuronal loss in the cortex of *nicastrin* cKO mice at 4.5 months of age.

### 3.4 The effect of DEX on synapses and dendrites in nicastrin cKO mice

**Fig. 4:**
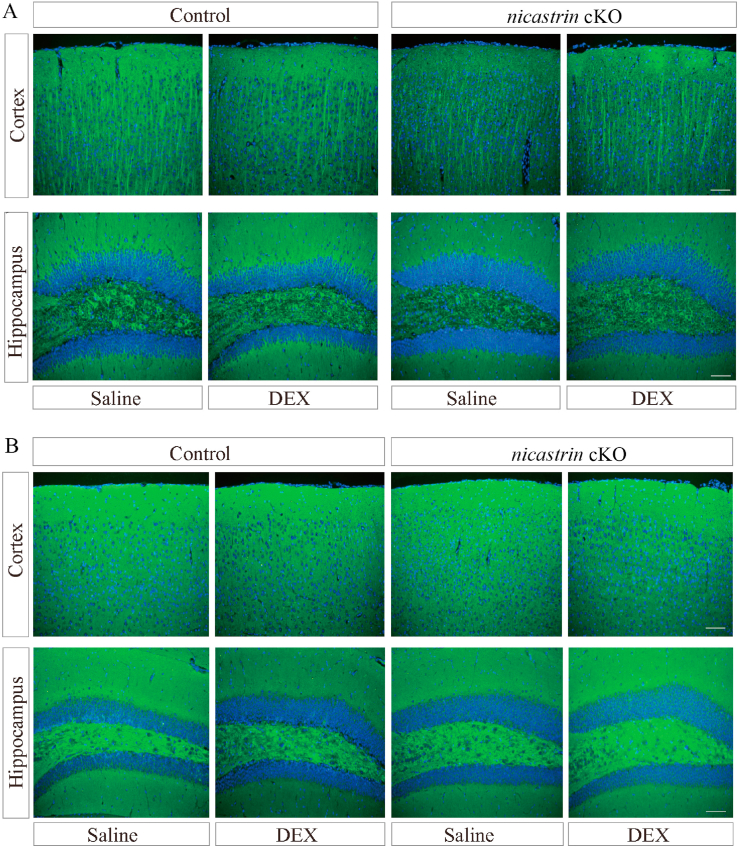
**The effect of DEX on the general morphology of synapses and dendrites in *nicastrin* cKO mice**. **A.** Immunofluorescence for MAP2 in the cortices and hippocampus of saline- or DEX-treated control and *nicastrin* cKO mice at 4.5 months. Scale bar = 50 μm. **B.** Immunofluorescence for SVP38 in the cortices and hippocampus of saline- or DEX-treated control and *nicastrin* cKO mice at 4.5 months. Scale bar = 50 μm.

To examine dendrites in *nicastrin* cKO mice, we first performed IHC on MAP2, a dendrite marker, using brain sections at 4.5 months. There was no detectable change on the MAP2 immuno-reactivity between control and *nicastrin* cKO cortices (Fig. 4A). Moreover, no difference was found on the MAP2 immuno-reactivity between saline-treated and DEX-treated *nicastrin* cKO mice. Second, we conducted IHC on SVP38, a marker for presynaptic terminals, but did not find difference on the SVP38 immuno-reactivity between control and *nicastrin* cKO mice (Fig. 4B). Overall, the general morphology of synapses and dendrites was not affected in *nicastrin* cKO mice.

## 4 Discussion

ND is a major threat to the elderly. However, there is no effective cure. It has been shown that neuroinflammation is an early pathological event in ND [29,37]. Thus, it is of great importance to screen anti-inflammation agents which may be beneficial for ND. To this end, we tested whether DEX, a potent drug to treat inflammation, could ameliorate neuroinflammation in an ND mouse model. We used DEX to treat *nicastrin* cKO mice at 2.5 months of age for 2 months. We show that 4.5-month-old *nicastrin* cKO mice exhibit significant gliosis. This observation is consistent with those reported in our previous studies [29,31]. In addition, we observe that two-month DEX treatment does not inhibit gliosis in the cortex of *nicastrin* cKO mice. These findings strongly suggest that DEX may not be a potential drug to treat neuroinflammation in ND.

It has been controversial whether DEX is beneficial to neuroinflammation in animal models. It was reported that DEX significantly reduce levels of several Borrelia burgdorferi (Bb)-induced immune mediators in culture supernatants of FC explants, astrocytes, microglia and oligodendrocytes [38]. In contrast, a recent study showed that chronic DEX exposure significantly increases apoptosis and causes neuron injury in rats hippocampal neurons [39]. Findings from clinical trials on non-steroidal anti-inflammation drugs (NSAID) are somehow controversial as well [17,40,41]. We reason that the discrepancy between our study and others may be due to different animal models and different dosage of DEX used. Indeed, the dosage of DEX may be an important factor to affect treatment effects. For example, it has been shown that DEX could prevent dopaminergic neuron loss in a mouse model of PD when the dosage is 10 but not 1 mg/kg [38]. Consistent with this finding, it is believed that high dosage of corticosteroids could reduce inflammatory cytokines [42]. In our study, the dosage of DEX was 10 mg/kg, which is equivalent to those reported by other groups [32]. Although the dosage of DEX used in this study significantly increases the body weight of the mice, it does not reduce or inhibit gliosis in *nicastrin* cKO mice. Taken together, this study suggests that DEX may not provide significant beneficial effects on neurodegenerative diseases.

## Funding

This work was supported by a grant from the Nanjing Key Medical Discipline (ZKX18014).

## CRediT authorship contribution statement

**Xiaolian Ye:** Investigation, Writing - original draft. **Gang Zou:** Software. **Jinxing Hou:** Investigation. **Huiru Bi:** Investigation. **Cuihua Zhou:** Formal analysis. **Runmin Wang:** Investigation. **Yun Xu:** Resources. **Chun Wang:** Data curation. **Guiquan Chen:** Methodology, Data curation. **Zhenyu Yin:** Project administration. **Jinping Zhang:** Supervision, Resources. **Chaoli Huang:** Writing - review & editing, Conceptualization.

## Declaration of competing interest

No potential conflicts of interest were disclosed.
